# Schwannoma-associated third nerve palsy: A pediatric case report

**DOI:** 10.1016/j.heliyon.2022.e09211

**Published:** 2022-03-30

**Authors:** Ashley A. Moeller, Louis A. Sokol, Chang Y. Ho

**Affiliations:** a Indiana University School of Medicine, Riley Hospital for Children, Indianapolis, IN, United States; b Indiana University, Bloomington, IN, United States

**Keywords:** Third nerve palsy, Oculomotor nerve palsy, Cranial nerve tumor, Schwannoma, Pediatric

## Abstract

Acquired third nerve palsies are infrequently seen in children, but are often associated with serious pathologies. This article presents a pediatric case of tumor-associated, isolated third nerve palsy, which took two years to diagnose. The patient initially presented with an isolated, dilated pupil and progressed over several months to a complete third nerve palsy. In this case, high-resolution neuroimaging eventually led to the diagnosis of a presumed schwannoma as the cause of her third nerve palsy. We review her case, the importance of high-resolution imaging, and management options.

## 1 Introduction

The third cranial nerve, also known as the oculomotor nerve, controls movement in four of the six major eye muscles. This allows elevation, depression, and adduction of the eye. The third nerve also aids in constricting the pupil, focusing the lens, and the ability to retract the eyelid. A complete third nerve palsy then features eye deviation in a “down and out” position, a dilated pupil, diplopia, and ptosis.

**Figure 1:**
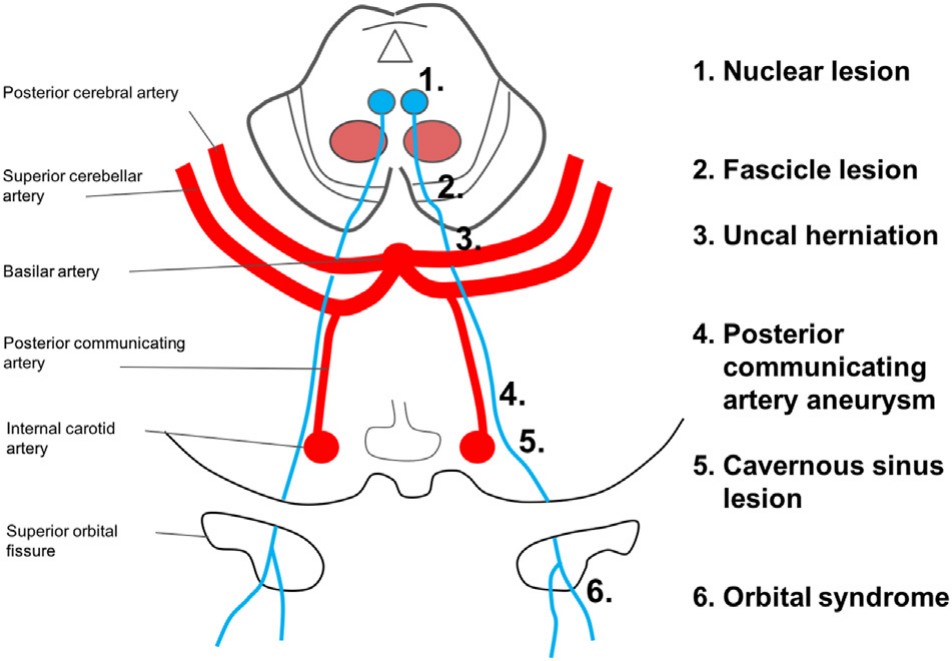
Anatomical course of the third cranial nerve and potential lesion sites. Reprinted with permission from Dr. Jonathan A. Micieli [9].

Localization of a lesion within the course of the third nerve can determine its cause (Figure 1). The nerve emanates from a midbrain nucleus, which is composed of multiple subnuclei. The medial rectus, inferior rectus, and inferior oblique muscles are supplied by their corresponding ipsilateral subnuclei, while the superior rectus muscle has contralateral supply. A single central caudal nucleus sends fibers to both the ipsilateral and contralateral levator palpebrae muscles. Therefore, a nuclear (brainstem) lesion typically results in complete ipsilateral third nerve palsy, along with bilateral superior rectus weakness and eyelid ptosis [1].

Upon exiting the brainstem, the third nerve travels lateral to the basilar artery and between the superior cerebellar (SCA) and posterior cerebral arteries (PCA). This is a common location for a compressive lesion, such as an aneurysm or uncal herniation. Compressive lesions cause pressure first on the parasympathetic fibers, which lie in the periphery of the nerve. The parasympathetic preganglionic fibers originate in the Edinger-Westphal nucleus in the midbrain. They then course in the periphery of the third nerve until synapsing in the orbital ciliary ganglion. The postganglionic fibers then travel to the sphincter pupillae, causing constriction of the pupil. Therefore, an isolated, dilated pupil is an indication of direct compression of the third nerve and can be the first sign of an aneurysm, tumor, or herniation. Pupil-sparing third nerve palsy is rare in children. In adults, it is largely secondary to microvascular ischemia in the setting of risk factors such as diabetes, hypertension, and smoking [1].

After passing between the SCA and PCA, the third nerve continues to course distally along the posterior communicating artery (PCOM) before entering the cavernous sinus. The PCOM is the most common location for an aneurysm to result in a third nerve palsy. The nerve then enters the cavernous sinus and travels along the superior aspect of the lateral wall. The trochlear nerve and the ophthalmic (V1) and maxillary (V2) divisions of the trigeminal nerve also travel along the lateral wall. Since the third nerve lies close to V1, a cavernous sinus lesion can sometimes present as painful ophthalmoplegia [1].

Exiting the cavernous sinus, the third nerve divides into superior and inferior branches. The branches then travel through the superior orbital fissure along with the trochlear nerve, abducens nerve, V1, and ophthalmic veins. A superior orbital fissure lesion, often caused by trauma, typically presents as a painful ophthalmoplegia with a dilated pupil, ptosis, and periorbital edema due to poor venous drainage [1].

We report a new case of pediatric third nerve palsy, the cause of which eluded us for two years. Only after repeated imaging using high-resolution MRI was the cause determined: cavernous sinus tumor. Management of such tumors, often determined to be schwannomas, is discussed.

## 2 Case report

A 3-year-old girl developed a painless left third nerve palsy. The onset of symptoms was staggered over several months, starting with dilation of the left pupil, followed by ptosis, and then limited movement of the left eye. Her initial ophthalmic examination yielded acuity of 20/20 in the right eye and 20/30 in the left eye. Her right pupil measured 3 ​mm and her left measured 5 ​mm, both reactive to light with no afferent pupillary defect. Her dilated funduscopic examination was unremarkable.

The patient's first MRI was completed when she initially presented with an isolated, dilated left pupil. The MRI was performed on a 1.5 T scanner with 3 ​mm thickness orbital cuts. There was no evidence of a lesion to account for her symptoms. Four months later she developed left eyelid ptosis and the MRI was repeated, but on a 3 T scanner with 2.5 ​mm thickness orbital cuts. Again, imaging was normal. Six months after initial presentation, she developed restricted elevation, depression, and adduction in the left eye. There was also a large left-sided exotropia and hypotropia on alignment testing and the pupil was no longer reactive to light. She had a complete third nerve palsy. The MRI was repeated again on the 3 T scanners, with orbital cuts at 2.5 mm thickness, but no lesions were detected. MR angiography and venography were also normal. Sedimentation rate, C-reactive protein, and lupus antibodies were unremarkable. CSF studies showed no evidence of an infectious, inflammatory, or demyelinating process.

Empiric treatment throughout the first year of her course included one month of oral steroids and four days of intravenous immunoglobulin. For two weeks, she was trialed on pyridostigmine for potential ocular myasthenia gravis. The patient had a persistent third nerve palsy which did not improve with any treatments.

**Figure 2:**
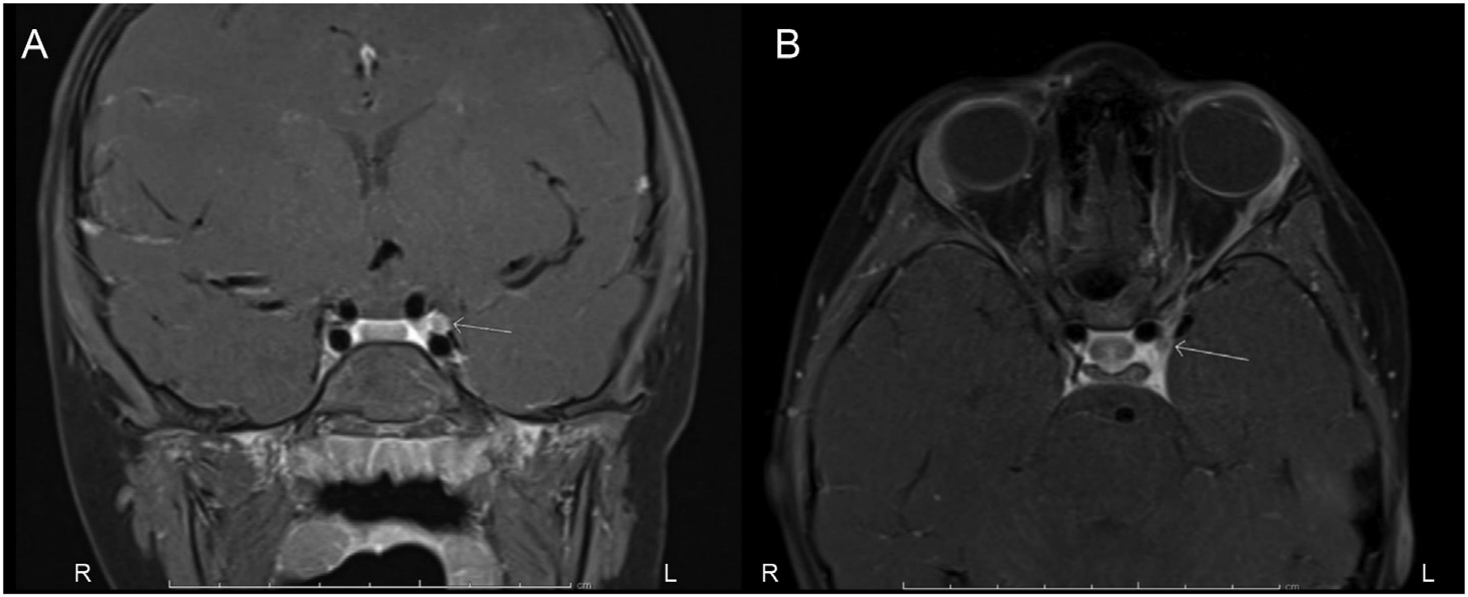
Coronal (A) and axial (B) high-resolution, fat-saturated, post-contrast T1 MRI showing a nodule of relative hypoenhancement compared to venous blood within the left cavernous sinus (arrows).

At the age of five, nearly two years after her initial presentation, an MRI was repeated on a 3 T scanner with 2.5 ​mm thickness cuts along the pathway of the oculomotor nerve. In addition, T2-weighted 3D-images were obtained with 1 ​mm thickness cuts of the orbits and oculomotor pathway. The cisternal portion of the nerves was normal in size, measuring 1.9 mm in diameter on the right and 1.8 mm on the left. However, on further tracing distally, there was a small nodular expansion of the left oculomotor nerve noted in the superior and lateral aspect of the left cavernous sinus. It was T1 hypoenhancing, when compared to the surrounding venous blood on post-contrast imaging, and measured 1.9 × 2.1 × 2.0 ​mm in transverse, anterior-posterior, and craniocaudal dimensions, respectively. It was best seen on axial fat-saturated, post-contrast T1 imaging (Figure 2) and was thought to be compatible with an oculomotor nerve schwannoma. Given that the cavernous sinus is partially bounded by dura, meningioma should also be considered. However, meningiomas classically demonstrate an enhancing dural tail that was not visualized in this case [2].

The patient is now six-years-old and the tumor has remained stable in size on repeat imaging. Her case has been discussed with multiple specialists who agree that intervention is not indicated at this time. She has undergone two strabismus surgeries with some improvement and is schedule for a third surgery soon.

## 3 Discussion

This is a case of a young girl who initially presented with an isolated, dilated left pupil, that gradually progressed to a complete third nerve palsy. This was most suggestive of a progressive compressive lesion affecting the peripheral third nerve. Since it was painless, it could be further localized to a lesion proximal to the superior orbital fissure and small enough in size to avoid impingement of V1. MRI utilizing high-resolution imaging of the oculomotor nerve on a 3 T scanner eventually led to the diagnosis of a presumed schwannoma in the left cavernous sinus. This highlights the importance of a dedicated, high-resolution MRI protocol for the detection of small cranial nerve tumors.

Schwannomas are peripheral nerve sheath tumors that grow very slowly. Pediatric cases of schwannoma in association with a third nerve palsy are rare and treatment is unfortunately limited [3]. Largely, tumor removal can make the cranial nerve palsy worse as nerve sacrifice cannot be avoided [4]. Therefore, unless the tumor shows aggressive growth, a more conservative approach of wait and watch is indicated. Gamma knife resection has shown mixed results for adults with third nerve palsy, but protocols for children are not yet readily available [5]. A minimally invasive surgery for oculomotor schwannomas, the endoscopic endonasal approach, has recently shown promise for adults but has not yet been attempted in children [6]. Decreased visual acuity, as seen in this patient, is often due to amblyopia. While ocular alignment can be improved with strabismus surgery, it rarely results in restoration of binocular alignment, and often has to be performed more than once [7]. Young children with tumor-associated third nerve palsy have the worst ophthalmologic outcomes [8]. Close, multidisciplinary involvement from neurologists, neuroradiologists, and ophthalmologists is required for optimal management of these challenging cases.

## Declarations

### Author contribution statement

All authors listed have significantly contributed to the investigation, development and writing of this article.

### Funding statement

This research did not receive any specific grant from funding agencies in the public, commercial, or not-for-profit sectors.

### Data availability statement

Data will be made available on request.

### Declaration of interests statement

The authors declare no conflict of interest.

### Additional information

No additional information is available for this paper.
